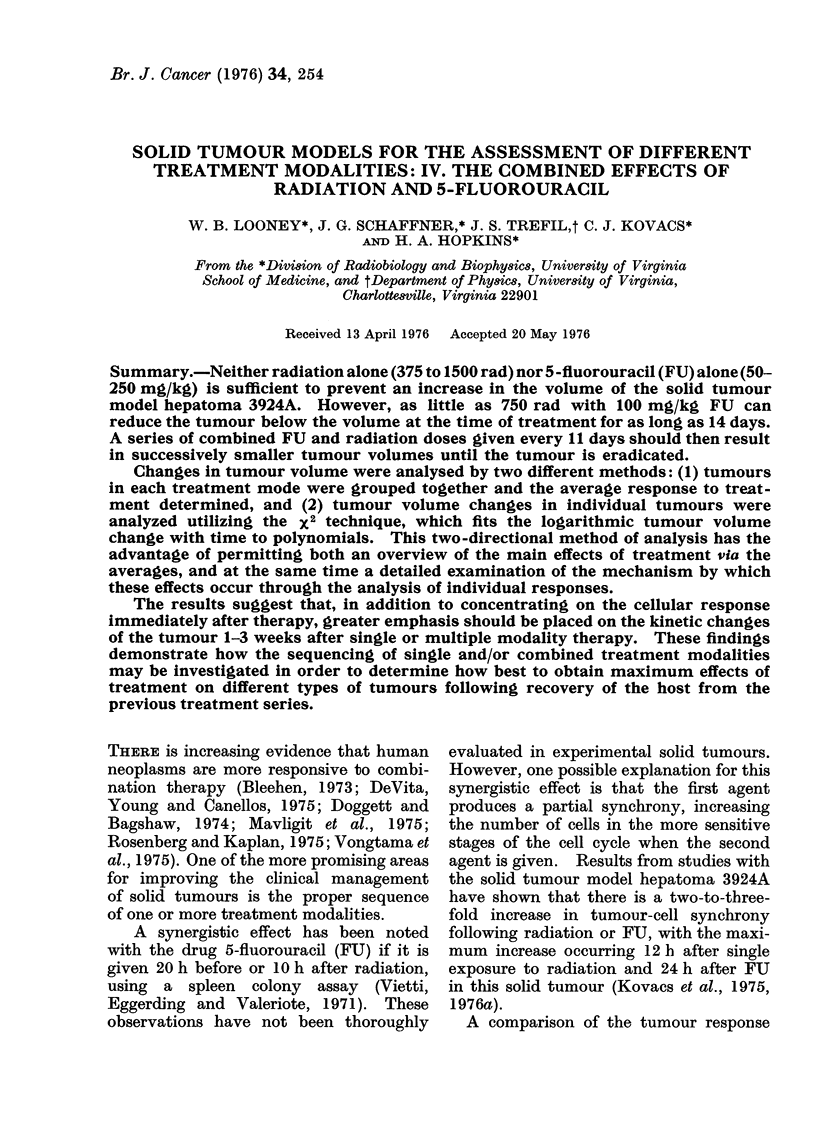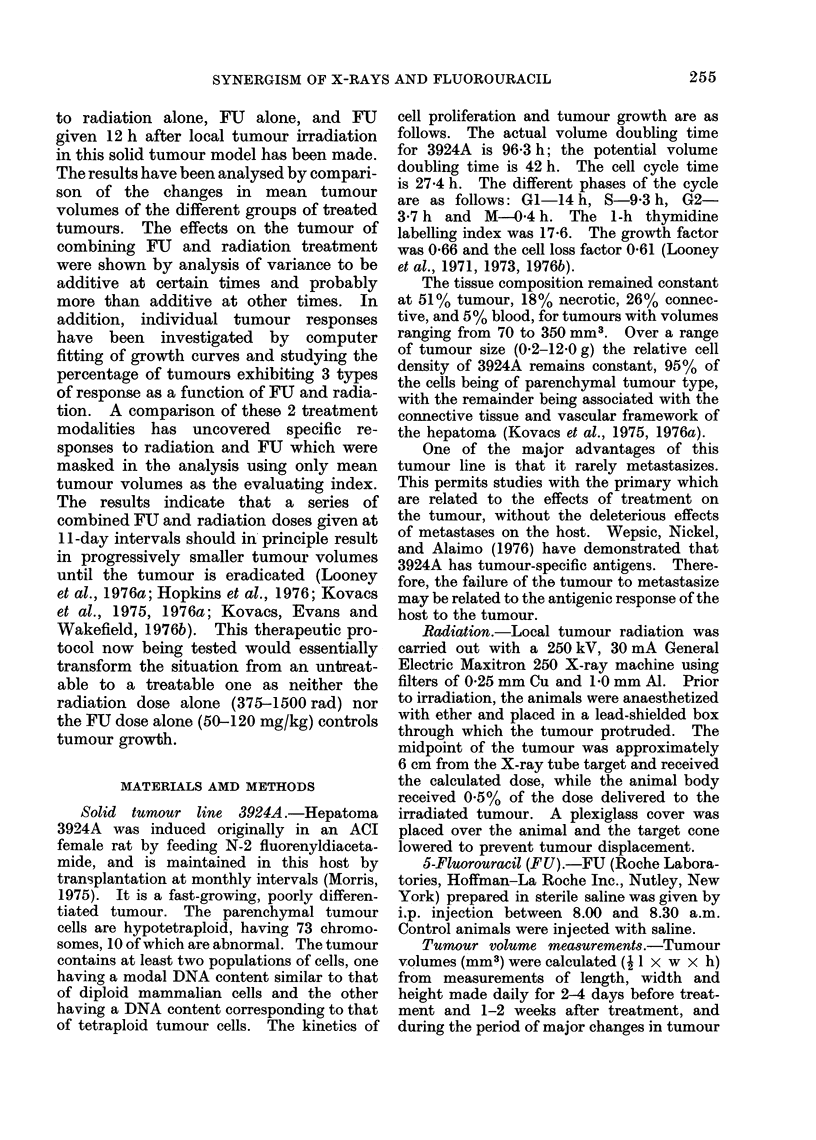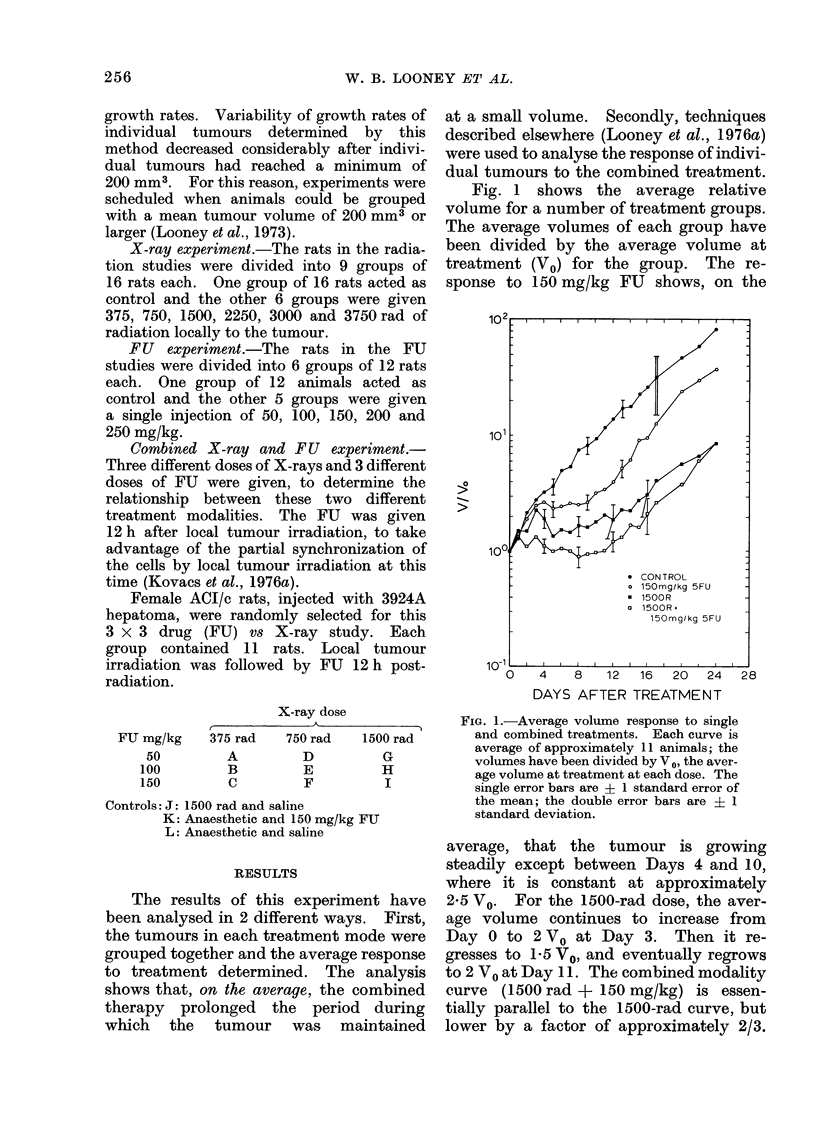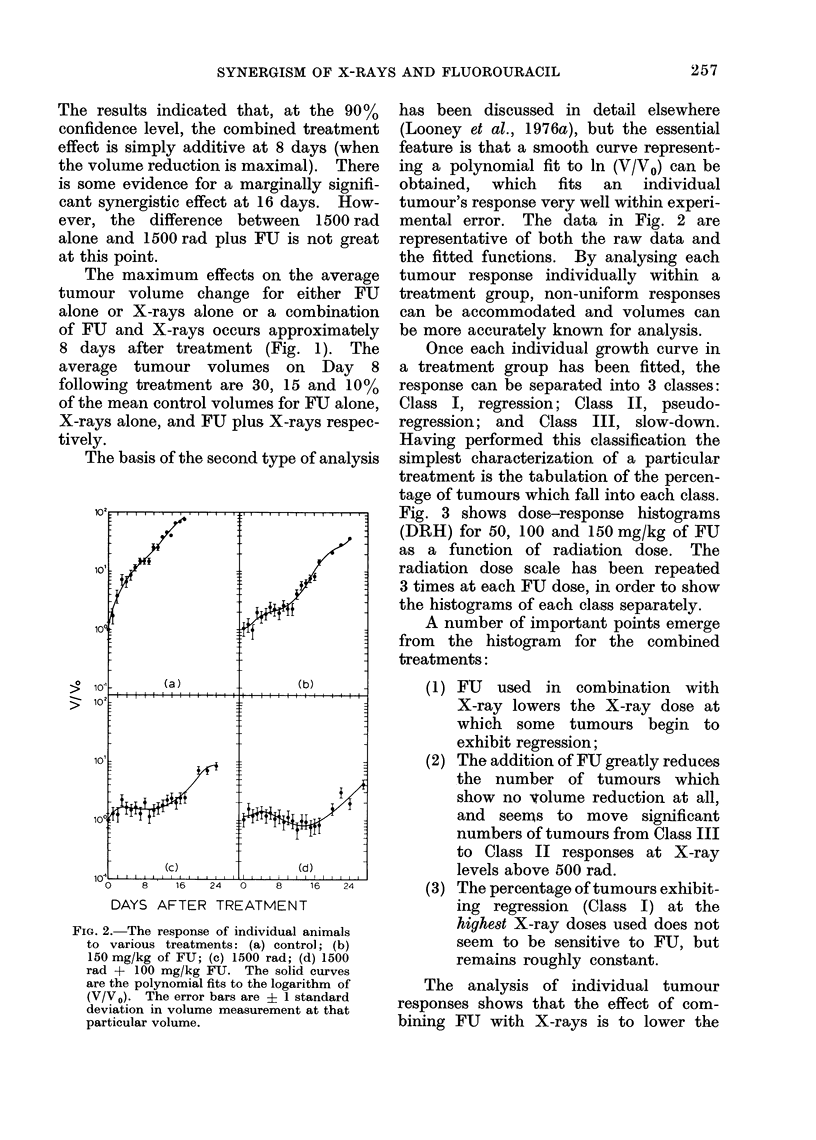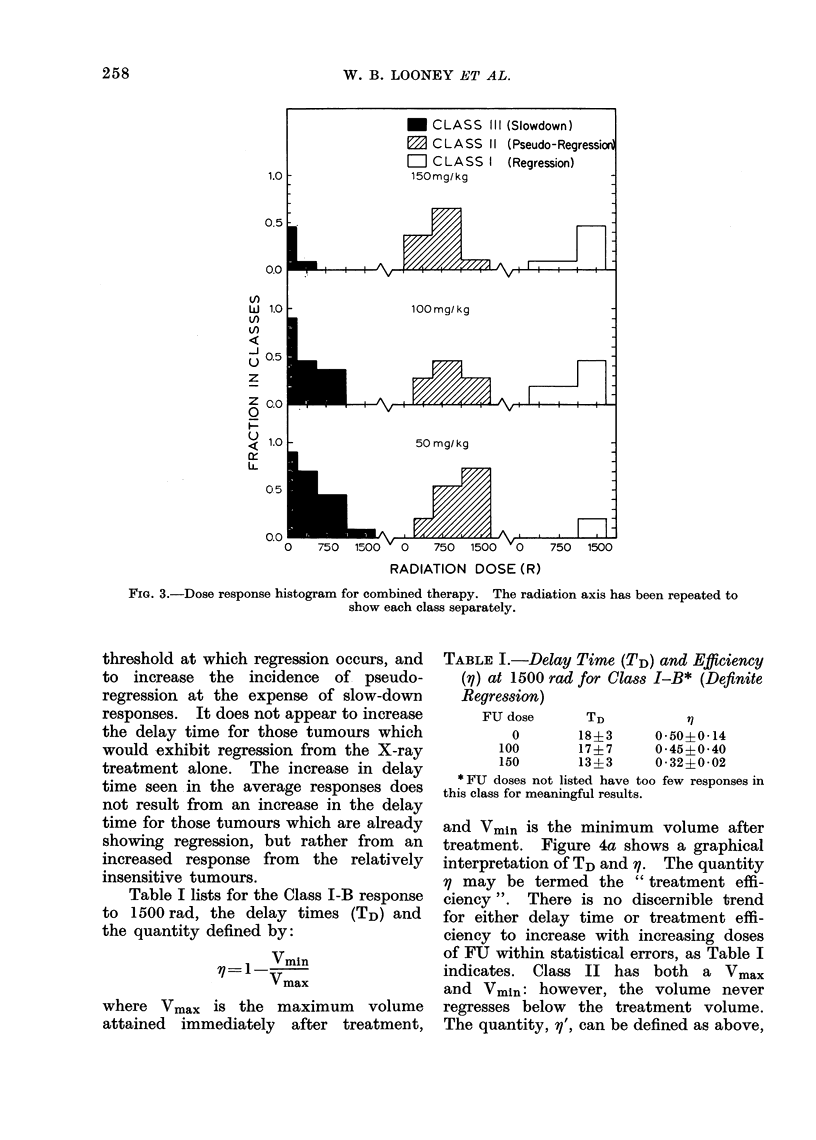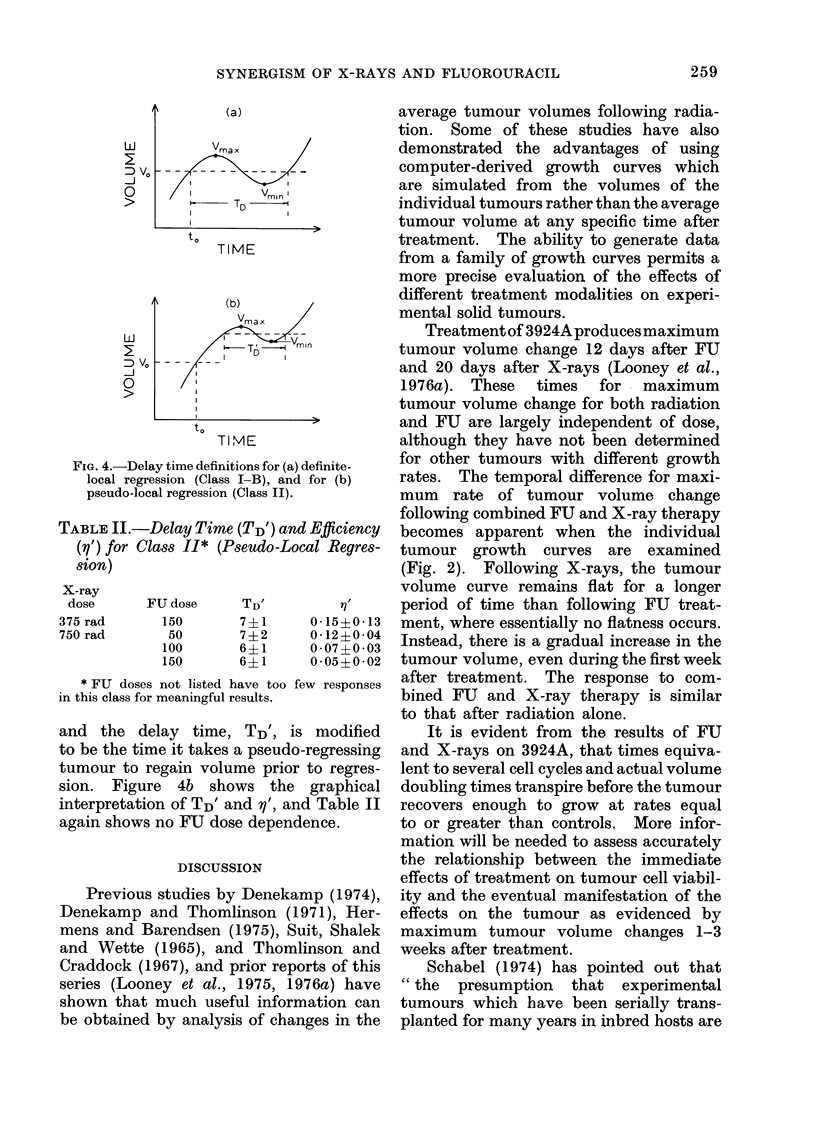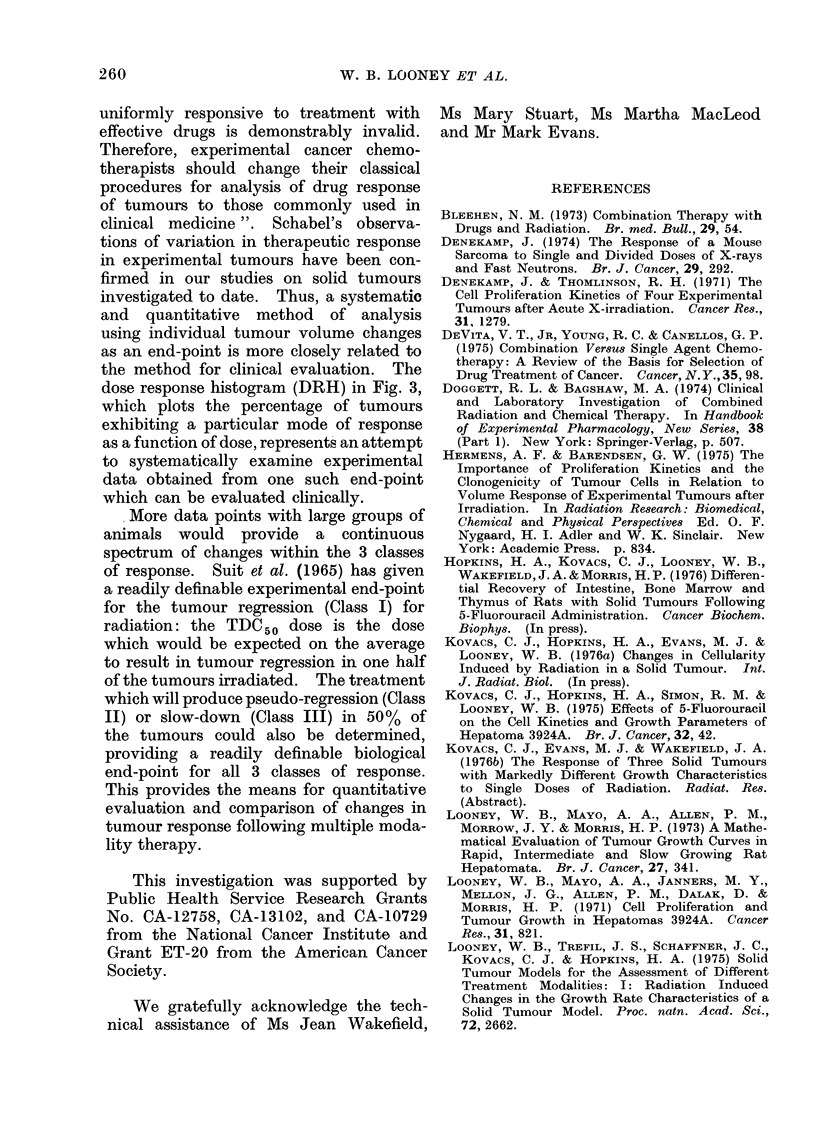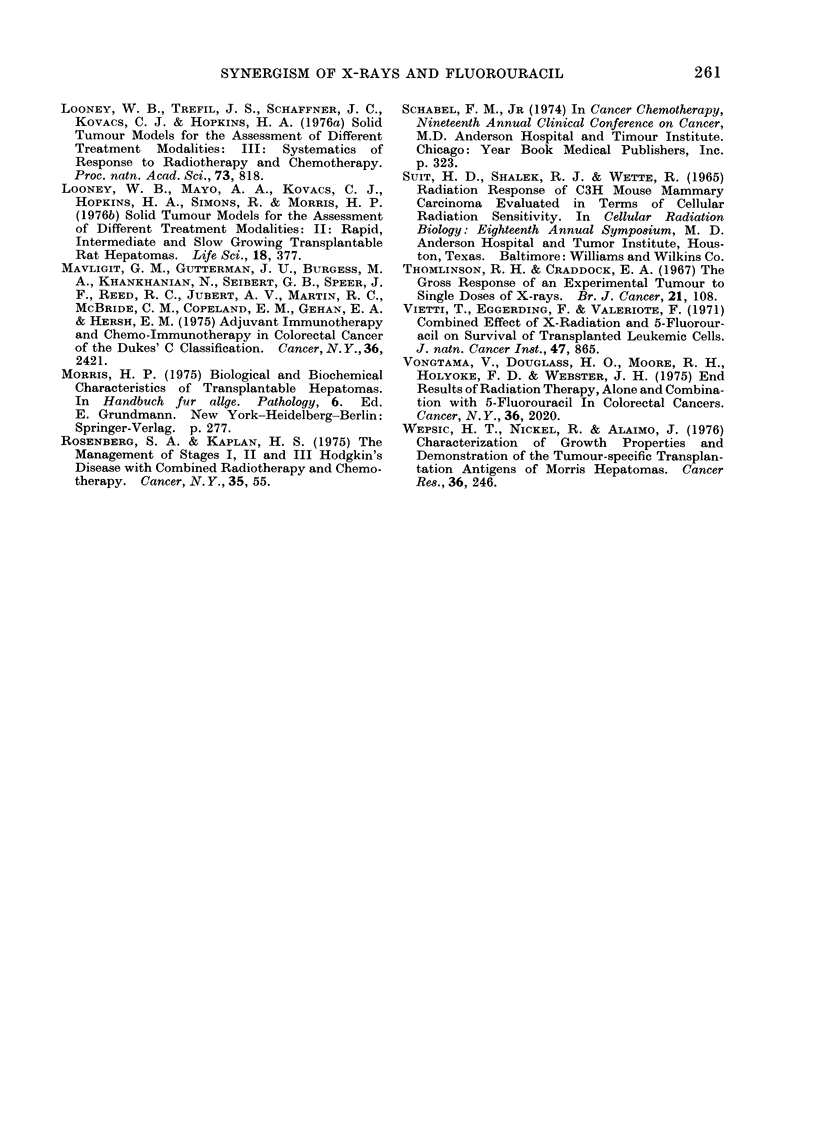# Solid tumour models for the assessment of different treatment modalities: IV. the combined effects of radiation and 5-fluorouracil.

**DOI:** 10.1038/bjc.1976.160

**Published:** 1976-09

**Authors:** W. B. Looney, J. G. Schaffner, J. S. Trefil, C. J. Kovacs, H. A. Hopkins

## Abstract

Neither radiation alone (375 to 1500 rad) nor5-fluorouracil (FU) alone (50-250 mg/kg) is sufficient to prevent an increase in the volume of the solid tumour model hepatoma 3924A. However, as little as 750 rad with 100 mg/kg FU can reduce the tumour below the volume at the time of treatment for as long as 14 days. A series of combined FU and radiation doses given every 11 days should then result in successively smaller tumour volumes until the tumour is eradicated. Changes in tumour volume were analysed by two different methods: (1) tumours in each treatment mode were grouped together and the average response to treatment determined, and (2) tumour volume changes in individual tumours were analyzed utilizing the chi2 technique, which fits the logarithmic tumour volume change with time to polynomials. This two-directional method of analysis has the advantage of permitting both an overview of the main effects of treatment via the averages, and at the same time a detailed examination of the mechanism by which these effects occur through the analysis of individual response. The results suggest that, in addition to concentrating on the cellular response immediately after therapy, greater emphasis should be placed on the kinetic changes of the tumour 1-3 weeks after single or multiple modality therapy. These findings demonstrate how the sequencing of single and/or combined treatment modalities may be investigated in order to detemine how best to obtain maximum effects of treatment on different types of tumours following recovery of the host from the previous treatment series.


*Br. J. Cancer* (1976) **34**, 254

### SOLID TUMOUR MODELS FOR THE ASSESSMENT OF DIFFERENT TREATMENT MODALITIES: IV. THE COMBINED EFFECTS OF RADIATION AND 5-FLUOROURACIL

W. B. LOONEY*, J. G. SCHAFFNER,* J. S. TREFIL,† C. J. KOVACS* AND H. A. HOPKINS*

*From the *Division of Radiobiology and Biophysics, University of Virginia School of Medicine, and †Department of Physics, University of Virginia, Charlottesville, Virginia 22901*

Received 13 April 1976 Accepted 20 May 1976

**Summary.—Neither radiation alone (375 to 1500 rad) nor 5-fluorouracil (FU) alone (50–250 mg/kg) is sufficient to prevent an increase in the volume of the solid tumour model hepatoma 3924A. However, as little as 750 rad with 100 mg/kg FU can reduce the tumour below the volume at the time of treatment for as long as 14 days. A series of combined FU and radiation doses given every 11 days should then result in successively smaller tumour volumes until the tumour is eradicated.**

**Changes in tumour volume were analysed by two different methods: (1) tumours in each treatment mode were grouped together and the average response to treatment determined, and (2) tumour volume changes in individual tumours were analyzed utilizing the $\chi^2$ technique, which fits the logarithmic tumour volume change with time to polynomials. This two-directional method of analysis has the advantage of permitting both an overview of the main effects of treatment *via* the averages, and at the same time a detailed examination of the mechanism by which these effects occur through the analysis of individual responses.**

**The results suggest that, in addition to concentrating on the cellular response immediately after therapy, greater emphasis should be placed on the kinetic changes of the tumour 1–3 weeks after single or multiple modality therapy. These findings demonstrate how the sequencing of single and/or combined treatment modalities may be investigated in order to determine how best to obtain maximum effects of treatment on different types of tumours following recovery of the host from the previous treatment series.**

THERE is increasing evidence that human neoplasms are more responsive to combination therapy (Bleehen, 1973; DeVita, Young and Canellos, 1975; Doggett and Bagshaw, 1974; Mavligit *et al.*, 1975; Rosenberg and Kaplan, 1975; Vongtama *et al.*, 1975). One of the more promising areas for improving the clinical management of solid tumours is the proper sequence of one or more treatment modalities.

A synergistic effect has been noted with the drug 5-fluorouracil (FU) if it is given 20 h before or 10 h after radiation, using a spleen colony assay (Vietti, Eggerding and Valeriote, 1971). These observations have not been thoroughly evaluated in experimental solid tumours. However, one possible explanation for this synergistic effect is that the first agent produces a partial synchrony, increasing the number of cells in the more sensitive stages of the cell cycle when the second agent is given. Results from studies with the solid tumour model hepatoma 3924A have shown that there is a two-to-three-fold increase in tumour-cell synchrony following radiation or FU, with the maximum increase occurring 12 h after single exposure to radiation and 24 h after FU in this solid tumour (Kovacs *et al.*, 1975, 1976*a*).

A comparison of the tumour response to radiation alone, FU alone, and FU given 12 h after local tumour irradiation in this solid tumour model has been made. The results have been analysed by comparison of the changes in mean tumour volumes of the different groups of treated tumours. The effects on the tumour of combining FU and radiation treatment were shown by analysis of variance to be additive at certain times and probably more than additive at other times. In addition, individual tumour responses have been investigated by computer fitting of growth curves and studying the percentage of tumours exhibiting 3 types of response as a function of FU and radiation. A comparison of these 2 treatment modalities has uncovered specific responses to radiation and FU which were masked in the analysis using only mean tumour volumes as the evaluating index. The results indicate that a series of combined FU and radiation doses given at 11-day intervals should in principle result in progressively smaller tumour volumes until the tumour is eradicated (Looney *et al.*, 1976*a*; Hopkins *et al.*, 1976; Kovacs *et al.*, 1975, 1976*a*; Kovacs, Evans and Wakefield, 1976*b*). This therapeutic protocol now being tested would essentially transform the situation from an untreatable to a treatable one as neither the radiation dose alone (375–1500 rad) nor the FU dose alone (50–120 mg/kg) controls tumour growth.

### MATERIALS AMD METHODS

*Solid tumour line 3924A*.—Hepatoma 3924A was induced originally in an ACI female rat by feeding N-2 fluorenyldiacetamide, and is maintained in this host by transplantation at monthly intervals (Morris, 1975). It is a fast-growing, poorly differentiated tumour. The parenchymal tumour cells are hypotetraploid, having 73 chromosomes, 10 of which are abnormal. The tumour contains at least two populations of cells, one having a modal DNA content similar to that of diploid mammalian cells and the other having a DNA content corresponding to that of tetraploid tumour cells. The kinetics of cell proliferation and tumour growth are as follows. The actual volume doubling time for 3924A is 96·3 h; the potential volume doubling time is 42 h. The cell cycle time is 27·4 h. The different phases of the cycle are as follows: G1—14 h, S—9·3 h, G2—3·7 h and M—0·4 h. The 1-h thymidine labelling index was 17·6. The growth factor was 0·66 and the cell loss factor 0·61 (Looney *et al.*, 1971, 1973, 1976*b*).

The tissue composition remained constant at 51% tumour, 18% necrotic, 26% connective, and 5% blood, for tumours with volumes ranging from 70 to 350 mm$^3$. Over a range of tumour size (0·2–12·0 g) the relative cell density of 3924A remains constant, 95% of the cells being of parenchymal tumour type, with the remainder being associated with the connective tissue and vascular framework of the hepatoma (Kovacs *et al.*, 1975, 1976*a*).

One of the major advantages of this tumour line is that it rarely metastasizes. This permits studies with the primary which are related to the effects of treatment on the tumour, without the deleterious effects of metastases on the host. Wepsic, Nickel, and Alaimo (1976) have demonstrated that 3924A has tumour-specific antigens. Therefore, the failure of the tumour to metastasize may be related to the antigenic response of the host to the tumour.

*Radiation*.—Local tumour radiation was carried out with a 250 kV, 30 mA General Electric Maxitron 250 X-ray machine using filters of 0·25 mm Cu and 1·0 mm Al. Prior to irradiation, the animals were anaesthetized with ether and placed in a lead-shielded box through which the tumour protruded. The midpoint of the tumour was approximately 6 cm from the X-ray tube target and received the calculated dose, while the animal body received 0·5% of the dose delivered to the irradiated tumour. A plexiglass cover was placed over the animal and the target cone lowered to prevent tumour displacement.

*5-Fluorouracil (FU)*.—FU (Roche Laboratories, Hoffman–La Roche Inc., Nutley, New York) prepared in sterile saline was given by i.p. injection between 8.00 and 8.30 a.m. Control animals were injected with saline.

*Tumour volume measurements*.—Tumour volumes (mm$^3$) were calculated ($\frac{1}{2}$ l × w × h) from measurements of length, width and height made daily for 2–4 days before treatment and 1–2 weeks after treatment, and during the period of major changes in tumour growth rates. Variability of growth rates of individual tumours determined by this method decreased considerably after individual tumours had reached a minimum of 200 $mm^3$. For this reason, experiments were scheduled when animals could be grouped with a mean tumour volume of 200 $mm^3$ or larger (Looney *et al.*, 1973).

*X-ray experiment.*—The rats in the radiation studies were divided into 9 groups of 16 rats each. One group of 16 rats acted as control and the other 6 groups were given 375, 750, 1500, 2250, 3000 and 3750 rad of radiation locally to the tumour.

*FU experiment.*—The rats in the FU studies were divided into 6 groups of 12 rats each. One group of 12 animals acted as control and the other 5 groups were given a single injection of 50, 100, 150, 200 and 250 mg/kg.

*Combined X-ray and FU experiment.*—Three different doses of X-rays and 3 different doses of FU were given, to determine the relationship between these two different treatment modalities. The FU was given 12 h after local tumour irradiation, to take advantage of the partial synchronization of the cells by local tumour irradiation at this time (Kovacs *et al.*, 1976*a*).

Female ACI/c rats, injected with 3924A hepatoma, were randomly selected for this 3 × 3 drug (FU) *vs* X-ray study. Each group contained 11 rats. Local tumour irradiation was followed by FU 12 h post-radiation.

| | X-ray dose | | |
|---|---|---|---|
| FU mg/kg | 375 rad | 750 rad | 1500 rad |
| 50 | A | D | G |
| 100 | B | E | H |
| 150 | C | F | I |

Controls: J: 1500 rad and saline
K: Anaesthetic and 150 mg/kg FU
L: Anaesthetic and saline

### RESULTS

The results of this experiment have been analysed in 2 different ways. First, the tumours in each treatment mode were grouped together and the average response to treatment determined. The analysis shows that, *on the average*, the combined therapy prolonged the period during which the tumour was maintained at a small volume. Secondly, techniques described elsewhere (Looney *et al.*, 1976*a*) were used to analyse the response of individual tumours to the combined treatment.

Fig. 1 shows the average relative volume for a number of treatment groups. The average volumes of each group have been divided by the average volume at treatment ($V_0$) for the group. The response to 150 mg/kg FU shows, on the average, that the tumour is growing steadily except between Days 4 and 10, where it is constant at approximately 2·5 $V_0$. For the 1500-rad dose, the average volume continues to increase from Day 0 to 2 $V_0$ at Day 3. Then it regresses to 1·5 $V_0$, and eventually regrows to 2 $V_0$ at Day 11. The combined modality curve (1500 rad + 150 mg/kg) is essentially parallel to the 1500-rad curve, but lower by a factor of approximately 2/3.

FIG. 1.—Average volume response to single and combined treatments. Each curve is average of approximately 11 animals; the volumes have been divided by $V_0$, the average volume at treatment at each dose. The single error bars are ± 1 standard error of the mean; the double error bars are ± 1 standard deviation.

The results indicated that, at the 90% confidence level, the combined treatment effect is simply additive at 8 days (when the volume reduction is maximal). There is some evidence for a marginally significant synergistic effect at 16 days. However, the difference between 1500 rad alone and 1500 rad plus FU is not great at this point.

The maximum effects on the average tumour volume change for either FU alone or X-rays alone or a combination of FU and X-rays occurs approximately 8 days after treatment (Fig. 1). The average tumour volumes on Day 8 following treatment are 30, 15 and 10% of the mean control volumes for FU alone, X-rays alone, and FU plus X-rays respectively.

The basis of the second type of analysis

FIG. 2.—The response of individual animals to various treatments: (a) control; (b) 150 mg/kg of FU; (c) 1500 rad; (d) 1500 rad + 100 mg/kg FU. The solid curves are the polynomial fits to the logarithm of $(V/V_0)$. The error bars are $\pm$ 1 standard deviation in volume measurement at that particular volume.

has been discussed in detail elsewhere (Looney *et al.*, 1976*a*), but the essential feature is that a smooth curve representing a polynomial fit to ln $(V/V_0)$ can be obtained, which fits an individual tumour's response very well within experimental error. The data in Fig. 2 are representative of both the raw data and the fitted functions. By analysing each tumour response individually within a treatment group, non-uniform responses can be accommodated and volumes can be more accurately known for analysis.

Once each individual growth curve in a treatment group has been fitted, the response can be separated into 3 classes: Class I, regression; Class II, pseudo-regression; and Class III, slow-down. Having performed this classification the simplest characterization of a particular treatment is the tabulation of the percentage of tumours which fall into each class. Fig. 3 shows dose–response histograms (DRH) for 50, 100 and 150 mg/kg of FU as a function of radiation dose. The radiation dose scale has been repeated 3 times at each FU dose, in order to show the histograms of each class separately.

A number of important points emerge from the histogram for the combined treatments:

(1) FU used in combination with X-ray lowers the X-ray dose at which some tumours begin to exhibit regression;
(2) The addition of FU greatly reduces the number of tumours which show no volume reduction at all, and seems to move significant numbers of tumours from Class III to Class II responses at X-ray levels above 500 rad.
(3) The percentage of tumours exhibiting regression (Class I) at the *highest* X-ray doses used does not seem to be sensitive to FU, but remains roughly constant.

The analysis of individual tumour responses shows that the effect of combining FU with X-rays is to lower the

FIG. 3.—Dose response histogram for combined therapy. The radiation axis has been repeated to show each class separately.

threshold at which regression occurs, and to increase the incidence of pseudo-regression at the expense of slow-down responses. It does not appear to increase the delay time for those tumours which would exhibit regression from the X-ray treatment alone. The increase in delay time seen in the average responses does not result from an increase in the delay time for those tumours which are already showing regression, but rather from an increased response from the relatively insensitive tumours.

Table I lists for the Class I-B response to 1500 rad, the delay times ($T_D$) and the quantity defined by:

$$\eta = 1 - \frac{V_{min}}{V_{max}}$$

where $V_{max}$ is the maximum volume attained immediately after treatment,

TABLE I.—*Delay Time* ($T_D$) *and Efficiency* ($\eta$) *at* 1500 *rad for Class I–B** (*Definite Regression*)

| FU dose | $T_D$ | $\eta$ |
|---|---|---|
| 0 | 18±3 | 0·50±0·14 |
| 100 | 17±7 | 0·45±0·40 |
| 150 | 13±3 | 0·32±0·02 |

* FU doses not listed have too few responses in this class for meaningful results.

and $V_{min}$ is the minimum volume after treatment. Figure 4*a* shows a graphical interpretation of $T_D$ and $\eta$. The quantity $\eta$ may be termed the " treatment efficiency ". There is no discernible trend for either delay time or treatment efficiency to increase with increasing doses of FU within statistical errors, as Table I indicates. Class II has both a $V_{max}$ and $V_{min}$: however, the volume never regresses below the treatment volume. The quantity, $\eta'$, can be defined as above,

FIG. 4.—Delay time definitions for (a) definite-local regression (Class I–B), and for (b) pseudo-local regression (Class II).

TABLE II.—*Delay Time* ($T_D'$) *and Efficiency* ($\eta'$) *for Class II** (*Pseudo-Local Regression*)

| X-ray dose | FU dose | $T_D'$ | $\eta'$ |
|---|---|---|---|
| 375 rad | 150 | 7±1 | 0·15±0·13 |
| 750 rad | 50 | 7±2 | 0·12±0·04 |
| | 100 | 6±1 | 0·07±0·03 |
| | 150 | 6±1 | 0·05±0·02 |

* FU doses not listed have too few responses in this class for meaningful results.

and the delay time, $T_D'$, is modified to be the time it takes a pseudo-regressing tumour to regain volume prior to regression. Figure 4*b* shows the graphical interpretation of $T_D'$ and $\eta'$, and Table II again shows no FU dose dependence.

### DISCUSSION

Previous studies by Denekamp (1974), Denekamp and Thomlinson (1971), Hermens and Barendsen (1975), Suit, Shalek and Wette (1965), and Thomlinson and Craddock (1967), and prior reports of this series (Looney *et al.*, 1975, 1976*a*) have shown that much useful information can be obtained by analysis of changes in the average tumour volumes following radiation. Some of these studies have also demonstrated the advantages of using computer-derived growth curves which are simulated from the volumes of the individual tumours rather than the average tumour volume at any specific time after treatment. The ability to generate data from a family of growth curves permits a more precise evaluation of the effects of different treatment modalities on experimental solid tumours.

Treatment of 3924A produces maximum tumour volume change 12 days after FU and 20 days after X-rays (Looney *et al.*, 1976*a*). These times for maximum tumour volume change for both radiation and FU are largely independent of dose, although they have not been determined for other tumours with different growth rates. The temporal difference for maximum rate of tumour volume change following combined FU and X-ray therapy becomes apparent when the individual tumour growth curves are examined (Fig. 2). Following X-rays, the tumour volume curve remains flat for a longer period of time than following FU treatment, where essentially no flatness occurs. Instead, there is a gradual increase in the tumour volume, even during the first week after treatment. The response to combined FU and X-ray therapy is similar to that after radiation alone.

It is evident from the results of FU and X-rays on 3924A, that times equivalent to several cell cycles and actual volume doubling times transpire before the tumour recovers enough to grow at rates equal to or greater than controls. More information will be needed to assess accurately the relationship between the immediate effects of treatment on tumour cell viability and the eventual manifestation of the effects on the tumour as evidenced by maximum tumour volume changes 1–3 weeks after treatment.

Schabel (1974) has pointed out that "the presumption that experimental tumours which have been serially transplanted for many years in inbred hosts are uniformly responsive to treatment with effective drugs is demonstrably invalid. Therefore, experimental cancer chemotherapists should change their classical procedures for analysis of drug response of tumours to those commonly used in clinical medicine ". Schabel's observations of variation in therapeutic response in experimental tumours have been confirmed in our studies on solid tumours investigated to date. Thus, a systematic and quantitative method of analysis using individual tumour volume changes as an end-point is more closely related to the method for clinical evaluation. The dose response histogram (DRH) in Fig. 3, which plots the percentage of tumours exhibiting a particular mode of response as a function of dose, represents an attempt to systematically examine experimental data obtained from one such end-point which can be evaluated clinically.

More data points with large groups of animals would provide a continuous spectrum of changes within the 3 classes of response. Suit *et al.* (1965) has given a readily definable experimental end-point for the tumour regression (Class I) for radiation: the $TDC_{50}$ dose is the dose which would be expected on the average to result in tumour regression in one half of the tumours irradiated. The treatment which will produce pseudo-regression (Class II) or slow-down (Class III) in 50% of the tumours could also be determined, providing a readily definable biological end-point for all 3 classes of response. This provides the means for quantitative evaluation and comparison of changes in tumour response following multiple modality therapy.

This investigation was supported by Public Health Service Research Grants No. CA-12758, CA-13102, and CA-10729 from the National Cancer Institute and Grant ET-20 from the American Cancer Society.

We gratefully acknowledge the technical assistance of Ms Jean Wakefield, Ms Mary Stuart, Ms Martha MacLeod and Mr Mark Evans.

### REFERENCES

BLEEHEN, N. M. (1973) Combination Therapy with Drugs and Radiation. *Br. med. Bull.*, **29**, 54.

DENEKAMP, J. (1974) The Response of a Mouse Sarcoma to Single and Divided Doses of X-rays and Fast Neutrons. *Br. J. Cancer*, **29**, 292.

DENEKAMP, J. & THOMLINSON, R. H. (1971) The Cell Proliferation Kinetics of Four Experimental Tumours after Acute X-irradiation. *Cancer Res.*, **31**, 1279.

DEVITA, V. T., JR, YOUNG, R. C. & CANELLOS, G. P. (1975) Combination *Versus* Single Agent Chemotherapy: A Review of the Basis for Selection of Drug Treatment of Cancer. *Cancer, N.Y.*, **35**, 98.

DOGGETT, R. L. & BAGSHAW, M. A. (1974) Clinical and Laboratory Investigation of Combined Radiation and Chemical Therapy. In *Handbook of Experimental Pharmacology, New Series*, **38** (Part 1). New York: Springer-Verlag, p. 507.

HERMENS, A. F. & BARENDSEN, G. W. (1975) The Importance of Proliferation Kinetics and the Clonogenicity of Tumour Cells in Relation to Volume Response of Experimental Tumours after Irradiation. In *Radiation Research: Biomedical, Chemical* and *Physical Perspectives* Ed. O. F. Nygaard, H. I. Adler and W. K. Sinclair. New York: Academic Press. p. 834.

HOPKINS, H. A., KOVACS, C. J., LOONEY, W. B., WAKEFIELD, J. A. & MORRIS, H. P. (1976) Differential Recovery of Intestine, Bone Marrow and Thymus of Rats with Solid Tumours Following 5-Fluorouracil Administration. *Cancer Biochem. Biophys.* (In press).

KOVACS, C. J., HOPKINS, H. A., EVANS, M. J. & LOONEY, W. B. (1976*a*) Changes in Cellularity Induced by Radiation in a Solid Tumour. *Int. J. Radiat. Biol.* (In press).

KOVACS, C. J., HOPKINS, H. A., SIMON, R. M. & LOONEY, W. B. (1975) Effects of 5-Fluorouracil on the Cell Kinetics and Growth Parameters of Hepatoma 3924A. *Br. J. Cancer*, **32**, 42.

KOVACS, C. J., EVANS, M. J. & WAKEFIELD, J. A. (1976*b*) The Response of Three Solid Tumours with Markedly Different Growth Characteristics to Single Doses of Radiation. *Radiat. Res.* (Abstract).

LOONEY, W. B., MAYO, A. A., ALLEN, P. M., MORROW, J. Y. & MORRIS, H. P. (1973) A Mathematical Evaluation of Tumour Growth Curves in Rapid, Intermediate and Slow Growing Rat Hepatomata. *Br. J. Cancer*, **27**, 341.

LOONEY, W. B., MAYO, A. A., JANNERS, M. Y., MELLON, J. G., ALLEN, P. M., DALAK, D. & MORRIS, H. P. (1971) Cell Proliferation and Tumour Growth in Hepatomas 3924A. *Cancer Res.*, **31**, 821.

LOONEY, W. B., TREFIL, J. S., SCHAFFNER, J. C., KOVACS, C. J. & HOPKINS, H. A. (1975) Solid Tumour Models for the Assessment of Different Treatment Modalities: I: Radiation Induced Changes in the Growth Rate Characteristics of a Solid Tumour Model. *Proc. natn. Acad. Sci.*, **72**, 2662.

Looney, W. B., Trefil, J. S., Schaffner, J. C., Kovacs, C. J. & Hopkins, H. A. (1976*a*) Solid Tumour Models for the Assessment of Different Treatment Modalities: III: Systematics of Response to Radiotherapy and Chemotherapy. *Proc. natn. Acad. Sci.*, **73**, 818.

Looney, W. B., Mayo, A. A., Kovacs, C. J., Hopkins, H. A., Simons, R. & Morris, H. P. (1976*b*) Solid Tumour Models for the Assessment of Different Treatment Modalities: II: Rapid, Intermediate and Slow Growing Transplantable Rat Hepatomas. *Life Sci.*, **18**, 377.

Mavligit, G. M., Gutterman, J. U., Burgess, M. A., Khankhanian, N., Seibert, G. B., Speer, J. F., Reed, R. C., Jubert, A. V., Martin, R. C., McBride, C. M., Copeland, E. M., Gehan, E. A. & Hersh, E. M. (1975) Adjuvant Immunotherapy and Chemo-Immunotherapy in Colorectal Cancer of the Dukes' C Classification. *Cancer, N.Y.*, **36**, 2421.

Morris, H. P. (1975) Biological and Biochemical Characteristics of Transplantable Hepatomas. In *Handbuch fur allge. Pathology*, **6**. Ed. E. Grundmann. New York–Heidelberg–Berlin: Springer-Verlag. p. 277.

Rosenberg, S. A. & Kaplan, H. S. (1975) The Management of Stages I, II and III Hodgkin's Disease with Combined Radiotherapy and Chemotherapy. *Cancer, N.Y.*, **35**, 55.

Schabel, F. M., Jr (1974) In *Cancer Chemotherapy, Nineteenth Annual Clinical Conference on Cancer*, M.D. Anderson Hospital and Timour Institute. Chicago: Year Book Medical Publishers, Inc. p. 323.

Suit, H. D., Shalek, R. J. & Wette, R. (1965) Radiation Response of C3H Mouse Mammary Carcinoma Evaluated in Terms of Cellular Radiation Sensitivity. In *Cellular Radiation Biology: Eighteenth Annual Symposium*, M. D. Anderson Hospital and Tumor Institute, Houston, Texas. Baltimore: Williams and Wilkins Co.

Thomlinson, R. H. & Craddock, E. A. (1967) The Gross Response of an Experimental Tumour to Single Doses of X-rays. *Br. J. Cancer*, **21**, 108.

Vietti, T., Eggerding, F. & Valeriote, F. (1971) Combined Effect of X-Radiation and 5-Fluorouracil on Survival of Transplanted Leukemic Cells. *J. natn. Cancer Inst.*, **47**, 865.

Vongtama, V., Douglass, H. O., Moore, R. H., Holyoke, F. D. & Webster, J. H. (1975) End Results of Radiation Therapy, Alone and Combination with 5-Fluorouracil In Colorectal Cancers. *Cancer, N.Y.*, **36**, 2020.

Wepsic, H. T., Nickel, R. & Alaimo, J. (1976) Characterization of Growth Properties and Demonstration of the Tumour-specific Transplantation Antigens of Morris Hepatomas. *Cancer Res.*, **36**, 246.